# Supplementary material for: The interactome of intact mitochondria by cross-linking mass spectrometry provides evidence for coexisting respiratory supercomplexes
Source: Mol Cell Proteomics. 2017 Dec 8;17(2):216–32. doi: 10.1074/mcp.RA117.000470 (PMC5795388; doi:10.1074/mcp.RA117.000470)
Supplement: Supplemental Data [file supp_RA117.000470_133922_0_supp_23978_fzffwf.zip › spectra_annotation/mito_spectra_annotation/460-1-1-1-9-1.pdf]

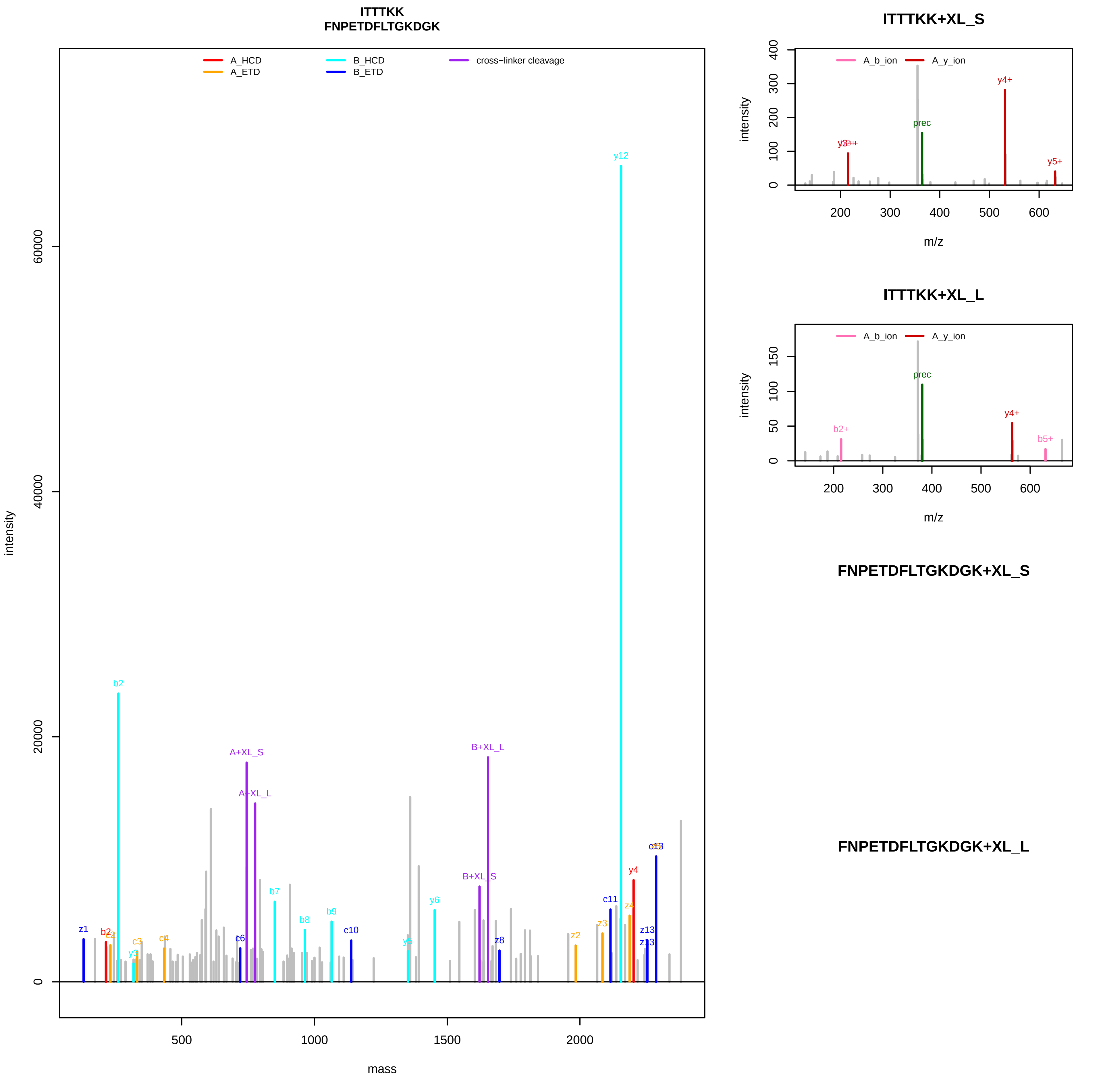

ITTTKK
FNPETDFLTGKDGK
A_HCD
A_ETD
B_HCD
B_ETD
cross−linker cleavage
intensity
mass
0
20000
40000
60000
500
1000
1500
2000
y12
b2
A+XL_S
A+XL_L
B+XL_L
B+XL_S
z1
b2
c2
c3
y3
c4
c6
b7
b8
b9
c10
y5
y6
z8
z2
z3
c11
z4
y4
z13
c13
ITTTKK+XL_S
A_b_ion
A_y_ion
y3++
prec
y4+
y5+
m/z
ITTTKK+XL_L
b2+
b5+
FNPETDFLTGKDGK+XL_S
FNPETDFLTGKDGK+XL_L
